# Investigation and Analysis of 108 Cases of Home Isolated Patients With Mild COVID-19

**DOI:** 10.1017/dmp.2020.296

**Published:** 2020-08-12

**Authors:** Hang Li, Yuan-Yuan Peng, Jin-Ping Lu

**Affiliations:** Department of Cardiology, Zhongnan Hospital, Wuhan University, Wuhan, P.R. China; Department of Geratology, Zhongnan Hospital, Wuhan University, Wuhan, P.R. China

**Keywords:** COVID-19, epidemic, home isolation, patient management

## Abstract

**Background:**

Coronavirus disease 2019 (COVID-19) began to spread across Wuhan, China, by the end of 2019, and patients were unable to be hospitalized because medical resources were limited.

**Methods:**

A questionnaire survey was conducted among 108 participants with mild COVID-19 who have isolated at home under the guidance of doctors. The results of the questionnaire and outpatient data were integrated to evaluate participants’ compliance with various epidemic prevention measures.

**Results:**

During isolation, most participants were able to follow epidemic prevention measures under the guidance of doctors. After 14 d from the start of isolation, 45.37% of the participants recovered. Approximately half of the participants were relieved of symptoms, and most of them were transferred to mobile cabin hospitals to continue isolation. Three participants with worsening symptoms were transferred to the designated hospitals. There were no deaths of the participants, but there were 7 family members that were infected.

**Conclusions:**

During a period of home isolation under the guidance of a doctor, individuals can comply with epidemic prevention measures and symptoms can be improved. Scientific home isolation may be an effective way to relieve the strain of medical and social resources during the epidemic of COVID-19.

From the end of 2019, coronavirus disease 2019 (COVID-19) has become widespread in China. COVID-19 is a respiratory infectious disease that can be spread through direct or indirect contact with droplets or aerosols, and there is no specific drug or vaccine to treat it.^1^

As of May 1, more than 84,000 confirmed cases and 4500 death cases have been documented in China. As of May 1, approximately 3,229,640 people were diagnosed with COVID-19 worldwide, and 229,888 people died.^2^ At present, the epidemic situation in Europe and America is very serious, especially in the United States, Italy, and Spain, and the number of new cases per day is still high. Due to the limited medical resources, a large number of suspected or confirmed patients cannot be admitted to the hospital.^3^

In the early stage of the outbreak, patients failed to receive timely medical treatment. The purpose of home isolation is to prevent the transmission of infection by diagnosed or potentially latent patients through physical isolation.^4^ In the early stages of the outbreak, there have been many close contacts with confirmed or suspected cases or cases that have been infected, and in latent period, home isolation is, therefore, an effective way to stop the transmission of the virus.^5^ Indeed, we found that, although many severe patients in the early stage were unable to receive timely treatment due to the limitation of medical resources, leading to deterioration of their conditions or even death, there were still many patient with mild symptoms gradually improved or even recovered through effective measures to limit transmission. Therefore, the study hypothesized that, in the early stages of the epidemic, under special circumstances in which hospital and social resources are insufficient, isolating patients with mild COVID-19 at home under the guidance of doctors may become an effective response measure.

## METHODS

### Ethical Approval

This study was conducted in accordance with the edicts of the 1975 *Declaration of Helsinki* and was approved by the Medical Ethics Committee, Zhongnan Hospital of Wuhan University (approval number: 2020040K). Participants were informed in writing and verbally about the study the voluntary nature of their participation, and their right to withdraw at any time without consequences. Moreover, anonymity and confidentiality were assured and observed. Participants were assigned identification codes, which were used to present data.

### Study Participants

This study included mild COVID-19 patients who have undergone home isolation under the guidance of doctors in the early stage of the epidemic (January 1 to February 8, 2020). The diagnosis and treatment standards of patients included in this study refer to the “Diagnosis and treatment plan of new coronavirus infection pneumonia (The Fifth Edition)” issued by the National Health Commission of the People’s Republic of China on February 4.^6^ The study included both adult individuals who obtained positive results for nucleic acids of severe acute respiratory syndrome coronavirus 2 (SARS-CoV-2) test and people who were confirmed by clinical symptoms and CT scans. All participants experienced mild symptoms (fever, cough, or lack of strength), and hospitalization was not required by doctors. Those with severe symptoms, previously diagnosed with serious cardiovascular (heart failure, myocardial infarction, cardiomyopathy, etc.) and cerebrovascular diseases and women during pregnancy or lactation were excluded from this study. All participants have been informed of the treatment measures by doctors from Zhongnan Hospital of Wuhan University on telephone or online from the beginning of isolation and continued interacting during the period of isolation.

### Study Design

A cross-sectional study design was used, including the demographic and sociological data for each participant and disease-related information. In this study, a questionnaire was designed and combined with the corresponding outpatient data of participants to evaluate the compliance with epidemic prevention measures during isolation and return visits for isolation.

### Measures to Limit Transmission in Home Isolation

During home isolation, all participants have been asked to comply with the following measures to limit transmission (Table 1).

**TABLE 1:** Anti-epidemic Measures in Home Isolation

Variable	Measure
Isolation environment	1. All participants should live in a well-ventilated single room or public area and avoid living in the same room with family members. In addition, participants should wear surgical masks or N95 masks in their daily lives
	2. Open the window for ventilation at least twice a day and 30 min each time to maintain the air flow in the bedroom, use 75% alcohol or 1000 mg/L chlorine-containing disinfectant to clean and wipe furniture in bedroom every day.
	3. Daily household items and toilet should be completely separated from other family members to avoid cross-infection, tableware used by our participants should be cleaned and disinfected separately.
	4. A regular caregiver is needed to take care of daily life of each participant (A caregiver should wear a disposable surgical mask or N95 mask when entering the isolation room).
Condition self-monitoring	1. Check body temperature frequently. The normal body temperature is below 37.3 °C. If possible, use the blood oxygen clip to monitor the finger pulse oxygen, normal blood oxygen saturation should be higher than 94%.
	2. If the body temperature exceeds 38 °C for 2 consecutive days, and symptoms of dyspnea, such as shortness of breath, breathlessness, and continuous decrease in blood oxygen saturation occur, it is necessary to visit the designated hospital right away.
	3. Caregivers monitor body temperature daily and seek medical attention if symptoms such as fever, cough, and diarrhea occur within 14 days of the last contact with the isolators.
Diet and drug guidance	1. According to the severity of symptoms (evaluated by doctor), the participants were asked to take Lianhua Qingwen granules (1 pack 3 times a day) for 5-10 days; oseltamivir phosphate capsules (75 mg twice a day) for 5 days; moxifloxacin hydrochloride tablets (0.4 g every day) for 6-12 days; some participants with obvious symptoms add arbidol tablets (0.2 g 3 times a dy) for 7-10 days as appropriate; when the body temperature returns to normal for 3 days and the cough symptoms are relieved, the drug can be stopped
	2. Participants should increase the amount of drinking water, about 2000 mL per day. For participants with fever, if the body temperature is lower than 38 °C, physical cooling should be given; if the body temperature ≥ 38.5 °C, take 1 tablet of paracetamol (325 mg).
	3. Ensure adequate energy intake, reasonable mix of fruits, vegetables, and meat, eat more high-quality protein foods to ensure adequate protein and vitamin intake.
Psychological counseling	1. During the isolation period, caregivers should create a good living environment to ensure normal circadian rhythm and eating habits
	2. While providing care, family members should also actively encourage participants, and help them to build their confidence in overcoming the disease.
	3. Prepare books or electronic products to distract them and relieve anxiety.
	4. Medical staff maintain regular online communication to understand the situation in time, carry out epidemic knowledge propaganda, and inform the importance of home isolation.

### Implementation of Home Isolation

The study questionnaire was designed to evaluate the implementation of the participant’s home isolation. The questionnaire includes the following questions: (Fully complied = F; Partially complied = P; Not complied = N) (the results are summarized in Table 2)

1. Did you keep living in a separate room during home isolation? (F/P/N).
2. Did you maintain daily ventilation during the isolation? (F/P/N).
3. Did you use separate toilets during isolation and disinfect them after each use? (F/P/N).
4. Did you keep your nose covered with tissue when coughing or sneezing during isolation? (F/P/N).
5. Did you wash your hands and disinfect them after contact with respiratory secretions (saliva, mucus)? (F/P/N).
6. Did you clean and disinfect your home daily as required? (F/P/N).
7. Did you maintain temperature monitoring every day? (F/P/N).
8. Did you wear a mask when you were in the public areas at home? (F/P/N).
9. Did you take medication according to the doctor’s advice during your isolation? (F/P/N).
10. Did you maintain a certain amount of daily activity/exercise? (F/P/N).
11. Did you keep regular contact with your doctor? (F/P/N).
12. Did you ever go out during isolation (F/P/N).
13. Is there any infection in your family members? (Yes/No).
14. Did your symptoms get worse during isolation? (Yes/No).
15. What is your status on the 14th day after isolation begins? (Recovery/isolation at home/In the mobile cabin hospitals/In the designated hospital).

**TABLE 2:** Implementation of Epidemic Prevention Measures

Investigation content	Fully Complied		Partially Complied		Not Complied	
	Number	%	Number	%	Number	%
Separate room living	102	94.44	6	5.56	0	0.00
Ventilation daily	104	96.30	4	3.70	0	0.00
Separate toilet, disinfected after use	24	22.22	10	9.26	74	68.52
Cover nose and mouth with tissue when coughing or sneezing	71	65.74	18	16.67	19	17.59
Disinfect hands after contacting respiratory secretion	81	75.00	15	13.89	12	11.11
Daily home disinfection	79	73.15	26	24.07	3	2.78
Monitoring body temperature	108	100.00	0	0.00	0	0.00
Wear masks during activities in public areas of the family	101	93.52	5	4.63	2	1.85
A constant caregiver	77	71.30	25	23.15	6	5.56
Take medicine according to the doctor’s instruction	108	100.00	0	0.00	0	0.00
Maintain moderate daily exercise	51	47.22	41	37.96	16	14.81
Regular contact with doctors	108	100.00	0	0.00	0	0.00
Prohibition of going out during quarantine	108	100.00		0.00		0.00

### Statistical Analysis

Data were presented as the median or the percentage (%). All data were analyzed by SPSS 22.0 software.

## RESULTS

### Study Participants

A total of 108 cases of home isolation participants were return visited, with the return of 108 valid questionnaires and the success rate of revisit was 100%. Among them, 58 participants were male and 50 were female, with a range of 20-82 years old and a median age of 49 years.

### Implementation of Home Isolation

Among the participants, more than 90% of them could fully comply with the rules, such as living in separate rooms, daily ventilation, temperature monitoring, wearing masks in the public areas of the family, taking medicine according to the doctor’s advice, and not going out, etc. However, they showed poor performance in using separate toilet and maintaining moderate daily exercise (Table 2).

### Participant’s Follow-up After Isolation

After 14 d of isolation, a total of 49 participants (45.37%) had negative results for nucleic acid tests. Among them, 36 were asymptomatic (fever, cough, dyspnea), 9 had mild cough, and 4 had mild cough combined with fatigue.

Forty-nine participants (45.37%) still had positive results for nucleic acid tests, but their symptoms improved. Among them, 14 participants had no obvious symptoms, but only showed fatigue and mild muscle aches, and continued to carry out home isolation; the remaining 35 participants (32.41%) with obvious symptoms were transferred to mobile cabin hospitals for subsequent isolation treatment.

Three participants (2.78%) were admitted to a designated hospital for treatment due to worsening symptoms (persistent fever, dyspnea) during isolation. No participant died, but 7 family members were infected.

### Study Limitations

The study methodology had limitations. For instance, the number of participants in our study is relatively small, and most of them are the young and the middle-aged. There are research reports that elderly COVID-19 patients face higher mortality and severe disease rate.^7^ In addition, underlying diseases of our participants may also have some impact on the outcome of COVID-19, which was not fully considered in this study, and, therefore, there might be some systematic errors.

## DISCUSSION

In the prevention and control of infectious diseases, the isolation of confirmed and suspected cases is an important step to stop the spread of the epidemic.^8^ The main purpose of this study was to investigate and analyze the compliance and the effects of measures to limit transmission of SARS-CoV-2 from mild COVID-19 patients during the period of relatively insufficient hospital resources in the early stage of the outbreak of the epidemic in Wuhan. The results showed that more than three-quarters of participants were able to observe and implement basic measures to limit transmission. Strict bans on going out, disinfection, and ventilation effectively prevent the transmission of the virus among people. However, some of the participants failed to cover their noses and mouths with tissues when coughing and sneezing, and a considerable number of participants failed to use separate toilets. According to the information obtained on the return visit, most participants have 2 separate toilets in their homes. We suspect that the inadequate implementation of the measures for patients using separate toilets may be related to their living habits (such as washing hands in public toilets, etc.). A significant number of participants failed to maintain adequate daily physical activity during isolation, which may be related to low moods and limited home space.

All participants in this study took oseltamivir phosphate capsules during home isolation; however, current study suggests that oseltamivir may not have the desired effect on COVID-19.^9^ Even so, after 14-d home quarantine, most of the participants’ viral nucleic acids turned negative, and their clinical symptoms were relieved (including participants whose nucleic acids had not yet turned negative), the remaining participants received appropriate treatment after a period of home isolation.

To date, many countries in the world have also seen a pandemic of COVID-2019. Some countries are still in the early stage, which is very similar to the early stage of Wuhan, facing a situation of short medical resources and public-health crisis.^10^ Our results showed that, for patients with mild symptoms, the measures to limit transmission under the guidance of professional doctors can play a role in comforting and isolating patients to a certain extent, which can be regarded as a good transitional way to deal with the shortage of hospital resources. Orderly home isolation under the management of doctors can greatly relieve the pressure of insufficient medical resources and social resources, and strive for valuable resources and time for the government to further control the epidemic.

## References

[1] Zhu N , Zhang D , Wang W , et al. A novel coronavirus from patients with pneumonia in China, 2019 N Engl J Med. 2020;382(8):727-733. doi: 10.1056/NEJMoa2001017 PMC7092803 31978945

[2] World Health Organization. Coronavirus disease (COVID-19) Situation Report-102. https://www.who.int/docs/default-source/coronaviruse/situation-reports/20200501-covid-19-sitrep.pdf?sfvrsn=742f4a18_4. Accessed May 1,2020.

[3] Tian H , Liu Y , Li Y , et al. An investigation of transmission control measures during the first 50 days of the covid-19 epidemic in China. Science. 2020;368(6491):638-642. doi: 10.1126/science.abb6105 32234804 PMC7164389

[4] Wang J , Liao Y , Wang X , et al. Incidence of novel coronavirus (2019-ncov) infection among people under home quarantine in Shenzhen, China. Travel Med Infect Dis. 2020:101660. doi: 10.1016/j.tmaid.2020.101660 PMC7270728 32247931

[5] Chiew CJ , Li Z , Lee VJ. Reducing onward spread of covid-19 from imported cases: Quarantine and ‘stay at home’ measures for travellers and returning residents to Singapore. J Travel Med. 2020;27(3):taaa049. doi: 10.1093/jtm/taaa049 PMC7184366 32297942

[6] National Health Commission of the People’s Republic of China. Diagnosis and treatment plan of new coronavirus infection pneumonia (The Fifth Edition). http://www.nhc.gov.cn/yzygj/s7653p/202002/3b09b894ac9b4204a79db5b8912d4440.shtml. Accessed February 1, 2020.

[7] Chen R , Liang W , Jiang M , et al. Risk factors of fatal outcome in hospitalized subjects with coronavirus disease 2019 from a nationwide analysis in China. Chest. 2020;158(1):97-105. doi: 10.1016/j.chest.2020.04.010 32304772 PMC7158802

[8] Bedford J , Enria D , Giesecke J , et al. Covid-19: towards controlling of a pandemic. Lancet. 2020;395(10229):1015-1018. doi: 10.1016/S0140-6736(20)30673-5 32197103 PMC7270596

[9] Sanders JM , Monogue ML , Jodlowski TZ , et al. Pharmacologic treatments for coronavirus disease 2019 (COVID-19): a review. JAMA. 2020. doi: 10.1001/jama.2020.6019 32282022

[10] Jakovljevic M , Bjedov S , Jaksic N , et al. Covid-19 pandemic and public and global mental health from the perspective of global health security. Psychiatr Danub. 2020;32(1):6-14. doi: 10.24869/psyd.2020.6 32303023

